# The convergent roles of metabolic dysfunction, alcohol, and iron in steatotic liver disease: a review

**DOI:** 10.1042/BSR20250106

**Published:** 2026-09-24

**Authors:** Afolabi Akanbi, Kim Bridle, Darrell H.G. Crawford

**Affiliations:** 1 Faculty of Health, Medicine and Behavioural Sciences, The University of Queensland, Brisbane, Australia; 2 Gallipoli Medical Research, Greenslopes Private Hospital, Brisbane, Australia

**Keywords:** Alcohol-associated liver disease (ALD), Fe-MetALD (Iron-associated metabolic and alcohol-related liver disease), Hepcidin, Iron overload, Metabolic dysfunction-associated steatotic liver disease (MASLD), MetALD

## Abstract

Steatotic liver disease (SLD) encompasses a broad spectrum of disorders influenced by metabolic dysfunction, alcohol use, and other cofactors. The updated classification of metabolic dysfunction-associated steatotic liver disease (MASLD) and alcohol-associated liver disease (ALD), along with the recognition of the overlapping entity MetALD, allows for better identification of patients at increased risk of progressive liver injury. While iron dysregulation is known to worsen both MASLD and ALD, its role within MetALD remains poorly defined. Understanding how iron contributes to this combined phenotype may provide new insight into why these patients experience more severe disease outcomes. This review synthesises current evidence linking iron to MASLD, ALD, and their intersection in MetALD. It introduces the concept of Fe-MetALD, a “triple-hit” phenotype where metabolic dysfunction, alcohol, and iron overload act together to accelerate liver injury. Mechanistically, these insults share common pathways involving oxidative stress, mitochondrial dysfunction, endoplasmic reticulum stress, inflammation, and hepatic stellate cell activation, collectively promoting fibrosis progression and potentially increasing the risk of cirrhosis and hepatocellular carcinoma. Experimental studies suggest that the combination of these insults produces more severe liver injury than any individual factor alone. Emerging therapies that target iron pathways, including hepcidin mimetics, ferroportin inhibitors, and iron-reduction strategies like venesection or chelation, may offer therapeutic potential, although current evidence remains largely preclinical. Recognising Fe-MetALD as a high-risk phenotype supports incorporating iron markers into diagnosis and developing therapies targeting metabolic, alcohol-related, and iron-mediated injury. Understanding this triple-hit interaction may improve risk stratification and guide future therapies for SLD.

## Introduction

**Figure 1:**
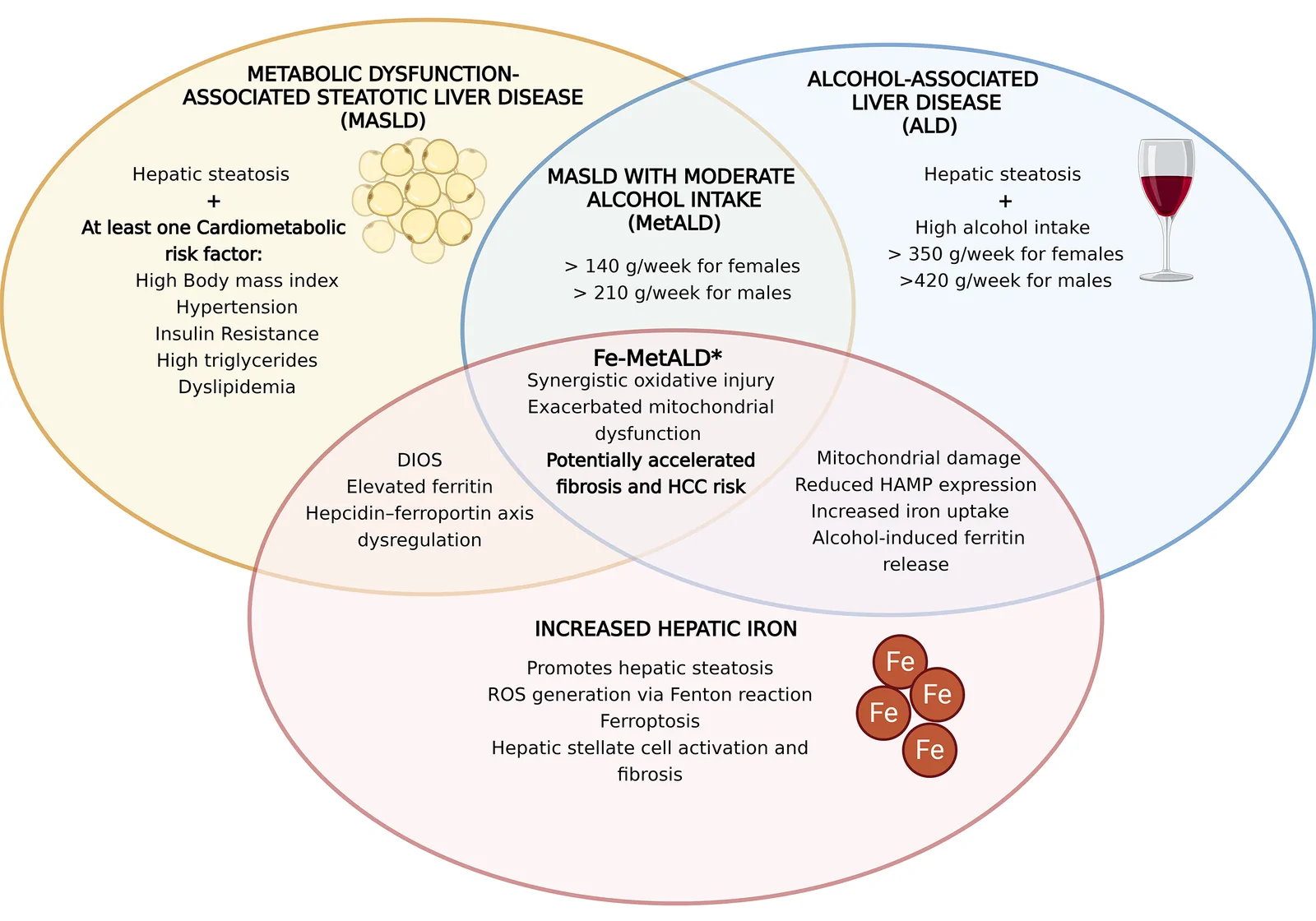
Pathophysiologic overlap of MASLD, ALD, and iron overload in SLD This Venn diagram illustrates the intersecting features of MASLD, ALD, and iron dysregulation. The central Fe-MetALD phenotype reflects co-toxic mechanisms from all three drivers. *The term reflects overlapping features of MASLD, alcohol intake, and iron overload based on emerging data and represents a proposed high-risk phenotype with synergistic pathogenesis.

Recent consensus has redefined fatty liver conditions under the umbrella of steatotic liver disease (SLD), replacing the old term non-alcoholic fatty liver disease (NAFLD) with metabolic dysfunction-associated steatotic liver disease (MASLD) [1]. MASLD is diagnosed when hepatic steatosis is present along with at least one of five cardiometabolic risk factors (see Figure 1). Importantly, the new nomenclature introduced MetALD (metabolic dysfunction and alcohol-associated liver disease) which describes patients who meet MASLD criteria and consume more than 140 g of alcohol per week for women (≈14 standard drinks) or 210 g/week for men [1]. MetALD identifies the overlap phenotype of metabolic dysfunction and significant alcohol intake that was not formally recognised in prior definitions. By contrast, alcohol-associated liver disease (ALD) remains a distinct category for those with harmful alcohol use without metabolic risk factors. This reclassification (finalised via a multi-society Delphi consensus in 2023) aimed to remove the “non-alcoholic” misnomer and stigma, better reflect pathogenesis, and ensure patients with dual aetiologies are recognised and included in research and clinical care [1].

Histologically, fatty liver caused by alcohol or metabolic dysfunction is almost indistinguishable, typically featuring steatosis, inflammation, hepatocyte ballooning, and fibrosis. The former distinction between NAFLD and ALD was largely based on an arbitrary alcohol consumption threshold [2], overlooking the fact that many individuals harbour risk factors from both pathways. Indeed, a recent reanalysis of UK Biobank data showed that 10.8% of individuals initially classified as MASLD have now been reclassified as MetALD under the new criteria [3]. In the United States, MetALD is estimated to affect 22–33 million people (of ∼80 million total SLD cases), a burden several-fold larger than pure alcohol-alone liver disease (∼5.9 million) [4,5]. Moderate alcohol intake, previously considered benign, has been shown to worsen outcomes in the presence of metabolic risk. While earlier studies suggested that light-to-moderate drinking might reduce non-alcoholic steatohepatitis (NASH) severity, more recent evidence indicates that no amount of alcohol is safe in the context of metabolic fatty liver disease [2].

**Figure 2:**
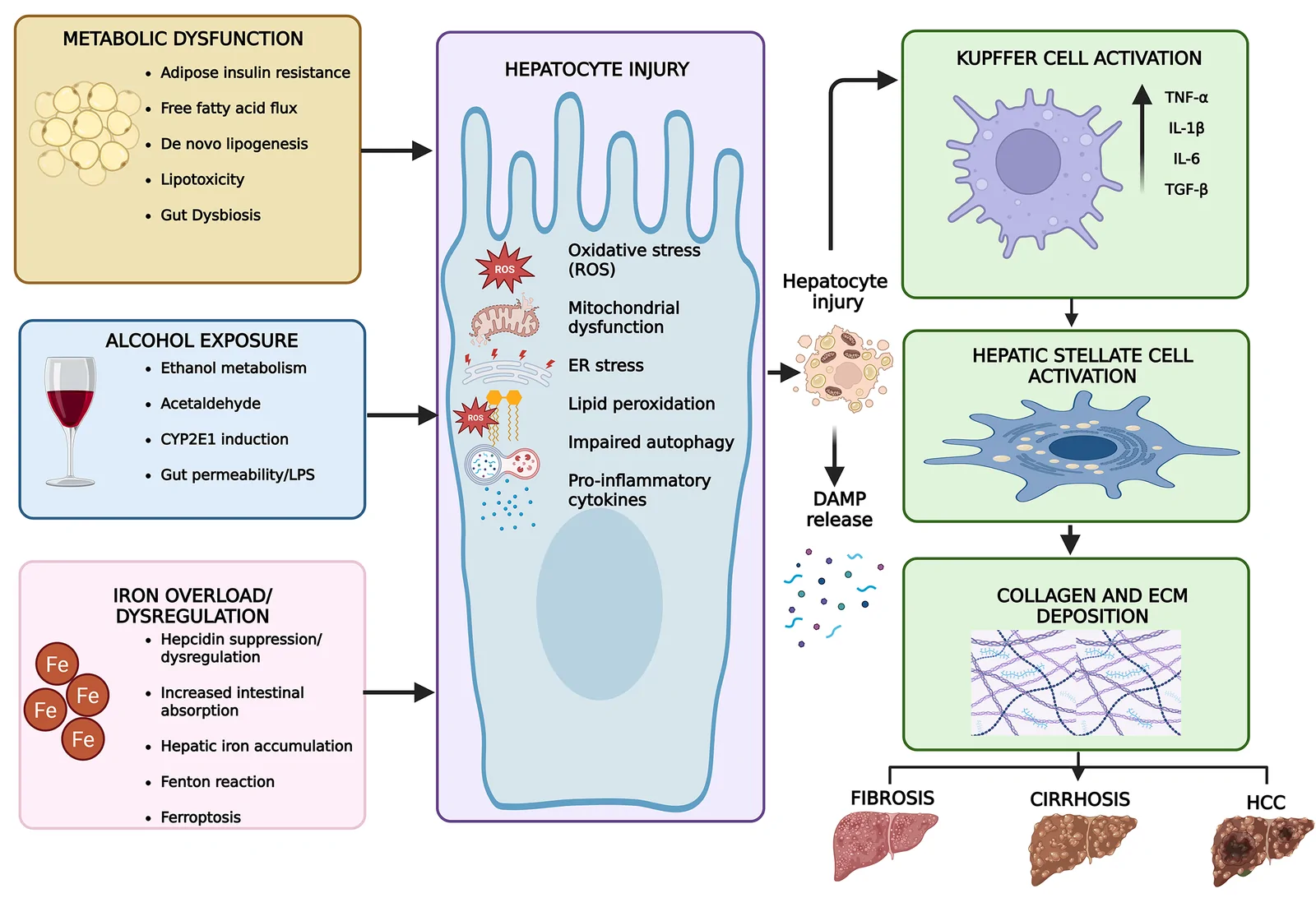
Integrated mechanistic framework of Fe-MetALD Metabolic dysfunction, chronic alcohol exposure, and iron overload/dysregulation converge through shared pathways, including oxidative stress, mitochondrial dysfunction, ER stress, lipid peroxidation, impaired autophagy, and pro-inflammatory cytokine signalling. These insults promote hepatocyte injury and DAMP release, leading to Kupffer cell activation, hepatic stellate cell activation, collagen and extracellular matrix deposition, and progressive fibrosis, cirrhosis, and HCC.

Iron is the most abundant trace metal in humans, and it is necessary for many biochemical processes; however, excess iron can be toxic to liver. Iron overload has emerged as an important cofactor in SLD, including MASLD, ALD, and their overlap (MetALD). In MASLD roughly one-third of patients exhibit hyperferritinemia with mild hepatic iron accumulation, often termed dysmetabolic iron overload syndrome (DIOS), which correlates with more severe steatohepatitis and fibrosis [6]. In ALD, iron deposition is even more common (seen in up to half of ALD cases) due to alcohol's suppression of hepcidin (encoded by HAMP), which increases intestinal iron absorption [7,8]. The resulting iron overload amplifies liver injury as the presence of hepatic iron in alcoholic cirrhosis approximately doubles the risk of death and significantly increases hepatocellular carcinoma (HCC) incidence. Patients with combined metabolic and alcohol risk factors (MetALD) likely experience similar or even heightened iron effects, though specific data on this subgroup are still emerging. The convergent mechanisms linking metabolic dysfunction, alcohol exposure and iron overload are summarised in Figure 2.

This review synthesises current knowledge on the role of iron in MASLD, ALD, and the newly defined MetALD. It explores epidemiological patterns, mechanistic pathways, and translational gaps, with particular emphasis on how the convergence of metabolic dysfunction, alcohol consumption, and iron overload synergistically accelerates liver injury, and identifies priorities for future research. A comparative summary of these disease entities is provided in Table 1.

**Table 1:** Clinical, pathological, and mechanistic comparison of MASLD, ALD, MetALD, and the proposed Fe-MetALD phenotype

Feature	MASLD	ALD	MetALD	Fe-MetALD
Definition	Hepatic steatosis with ≥1 cardiometabolic risk factor	Hepatic steatosis due to excessive alcohol consumption	MASLD with moderate alcohol intake	Proposed phenotype combining metabolic dysfunction, alcohol exposure and iron overload
Primary driver	Metabolic dysfunction	Alcohol consumption	Metabolic dysfunction + alcohol	Metabolic dysfunction + alcohol + iron overload
Alcohol consumption threshold	Below MetALD range	>350 g/week (female); >420 g/week (male)	140–350 g/week (female); 210–420 g/week (male)	Variable
Major risk factors	Obesity, T2DM, insulin resistance, dyslipidaemia	Chronic alcohol exposure	Combined metabolic and alcohol risk factors	Metabolic dysfunction, alcohol exposure, iron overload
Hepatic steatosis	Predominantly macro vesicular	Predominantly macro vesicular	Predominantly macro vesicular	Often accentuated steatosis [87].
Hepatocyte ballooning	Common	Common	Common	Enhanced by oxidative stress
Lobular inflammation	Present	Present, often neutrophil-rich	Present	Increased inflammatory activity [167]
Mallory-Denk bodies	May occur	Characteristic feature	Frequently observed	Can be present
Fibrosis pattern	Perisinusoidal/pericellular	Pericentral/pericellular	Mixed pattern	Accelerated fibrosis progression [87]
Iron deposition	DIOS; hepatocellular and/or reticuloendothelial	Hepatocytes and Kupffer cells	Variable	Potentially hepatocellular and Kupffer cell iron accumulation
Hepcidin status	Dysregulated	Suppressed by alcohol	Potentially dysregulated	Likely profoundly dysregulated
Oxidative stress	Increased lipid peroxidation	Increased CYP2E1-mediated ROS	Synergistically increased [3]	Further amplified through Fenton chemistry [78]
Major pathogenic mechanisms	Lipotoxicity, insulin resistance, inflammation	Acetaldehyde toxicity, CYP2E1, ROS	Combined metabolic and alcohol injury	Triple-hit convergence of metabolic dysfunction, alcohol, and iron
HCC risk	Moderate	Moderate	Increased [144]	Potentially high-risk phenotype [167]
Therapeutic approaches	Lifestyle modification, GLP-1 agonists, THR-β agonists	Alcohol abstinence, nutritional support	Weight loss and alcohol cessation	Weight loss, alcohol cessation and emerging iron-directed therapies
Current evidence level	Established	Established	Emerging clinical entity	Emerging/limited evidence

Abbreviations: ALD, alcohol-associated liver disease; DIOS, dysmetabolic iron overload syndrome; Fe-MetALD, iron-associated metabolic dysfunction-associated alcohol-related liver disease; HCC, hepatocellular carcinoma; MASLD, metabolic dysfunction-associated steatotic liver disease; MetALD, metabolic dysfunction-associated and alcohol-related liver disease; T2DM, type 2 diabetes mellitus.

### Overview of iron metabolism

Iron is the most abundant trace metal in humans, with approximately 3.5–5 grams found in healthy adults [9]. Iron is necessary for many biochemical processes, including gene regulation, electron transfer reactions, regulation of cell growth and differentiation, and binding and transport of oxygen [10]. Thus, humans require an abundant and consistent supply of dietary iron to maintain normal health. Iron homeostasis requires a complex and highly sophisticated set of regulatory approaches to meet the demands of cells as well as prevent excess accumulation [11]. Disorders of iron metabolism have serious consequences. Iron deficiency anaemia is the most common nutrient deficiency disorder in the world [12]. In contrast, iron overload is often a result of excess intestinal iron absorption [11,13].

Iron absorption and storage are tightly regulated, and several proteins play key roles in regulating mammalian iron homeostasis. These proteins are involved in controlling the rate of iron absorption, its recycling by reticuloendothelial cells, its delivery, utilisation, and storage by metabolically active tissues.

#### Iron metabolism proteins

Approximately 1–2 mg of iron is absorbed daily from dietary sources, primarily in the duodenum [9]. Dietary iron exists in two main forms: haem and non-haem iron. Divalent metal transporter (DMT1), ferroportin (FPN), and transferrin receptors (TFR) are key proteins responsible for iron transport across cellular membranes. Non-haem iron, found predominantly in plant-based foods, is less readily absorbed than haem iron. DMT1 plays a key role in its absorption by transporting ferrous iron across the apical membrane of enterocytes [14]. In contrast, haem iron is found predominantly in animal products and is more readily absorbed. Following intestinal uptake, intracellular haem can be catabolised by haem oxygenase to release ferrous iron [15].

FPN, previously known as iron-regulated transporter 1 (IREG1) [16], is essential in maintaining iron homeostasis, as it is the only identified iron efflux protein in the body [17]. FPN is expressed in all cells capable of releasing iron and is specifically involved in iron release into circulation from enterocytes, hepatocytes, iron-recycling macrophages and the placental syncytiotrophoblast. HAMP, the gene encoding hepcidin, controls systemic iron availability by binding FPN, inhibiting iron export and promoting FPN internalisation and degradation [17]. TFR1 is widely expressed and mediates transferrin-bound iron uptake in most cells, particularly erythroid precursors, whereas TFR2 is expressed predominantly in hepatocytes and erythroid cells [18]. As expected, the duodenal mRNA expression of these iron transport proteins (DMT1, FPN, TFR1) in mice increased when fed an iron-deficient diet and decreased following iron supplementation [19].

Ferritins are the main iron storage molecules, as approximately 25% of the iron in the body is stored as ferritin and haemosiderin (considered to be a degradation product of ferritin) [20]. The quaternary structure of ferritin consists of 24 subunits with tissue-specific and condition-dependent ratios of ferritin heavy chains and ferritin light chains. L-rich ferritins store iron for a prolonged period, and they have higher average iron content (>1500 Fe atoms/molecule) compared with H-rich ferritins (<1000 Fe atoms/molecule) [21].

Systemic iron sensing is largely regulated through the hepatic BMP-SMAD/hepcidin pathway. BMP2 and BMP6 signal through hemojuvelin (HJV) and BMP receptors to activate SMAD1/5/8-SMAD4 signalling and induce expression of hepcidin [22,23]. HFE and transferrin receptor 2 (TFR2) contribute to iron sensing and promote hepcidin expression [24], while TMPRSS6 (matriptase-2) negatively regulates this pathway [25]. During increased erythropoiesis, erythroferrone suppresses hepcidin production [26]. HFE interacts with both TFR1 and TFR2, although these interactions have distinct roles in iron sensing. Increasing holo-transferrin displaces HFE from TFR1, while TFR2 contributes to iron-responsive hepcidin regulation [27,28]. Loss of functional HFE or HJV results in inadequate hepcidin production and systemic iron overload [23]. In contrast, loss of TMPRSS6 causes inappropriately high hepcidin levels and iron-restricted erythropoiesis [29].

Dysregulation of iron homeostasis, whether due to genetic factors, altered absorption, or systemic metabolic disturbances, has been increasingly recognised as a key contributor to liver disease pathogenesis, including MASLD.

### Metabolic dysfunction-associated steatotic liver disease

MASLD, formerly known as NAFLD, is defined by the presence of at least one of the five cardiometabolic risk factors in the context of hepatic steatosis [30]. The term MASLD was introduced to better reflect the underlying metabolic dysfunction and to move away from a definition based on alcohol consumption exclusion. The disease spectrum ranges from simple hepatic steatosis to metabolic dysfunction-associated steatohepatitis (MASH), previously termed NASH, which can progress to fibrosis, cirrhosis, and HCC. The increasing prevalence of MASLD poses a significant public health challenge due to its association with cardiovascular disease (CVD). There is evidence from large cohort studies that indicates that MASLD is an independent risk factor for CVD morbidity and mortality [31–33].

#### Pathogenesis

The pathogenesis of MASLD is multifactorial, involving complex interactions between genetic, environmental, and metabolic factors. Genetic predisposition could influence susceptibility to MASLD, as it can cause alterations in molecular pathways in liver cells, such as glycolysis, export of triglycerides, intrahepatic lipolysis, and mitochondrial oxidation [34–36]. Genetic variants such as PNPLA3 I148M (rs738409) are strongly associated with hepatic fat accumulation and progressive liver injury [37,38], and are considered key determinants of disease severity in both paediatric and adult populations [39,40]. Additional findings have revealed that a loss-of-function variant in *HSD17B13* is associated with a reduced risk of chronic liver disease and progression from steatosis to steatohepatitis [41], while impaired *TM6SF2* function causally contributes to liver disease progression [42].

The gut microbiome, a complex community of microorganisms residing in the gastrointestinal tract, plays a pivotal role in maintaining metabolic and immune homeostasis. Emerging evidence suggests that alterations in the composition and function of the gut microbiota, termed dysbiosis, are intricately linked to the pathogenesis of MASLD [43,44]. In individuals with MASLD, dysbiosis often manifests as a reduced diversity of gut microbiota and an altered Firmicutes-to-Bacteroidetes ratio [45,46]. These changes can lead to increased intestinal permeability, allowing bacterial endotoxins such as lipopolysaccharides to translocate into the portal circulation. The influx of these endotoxins into the liver triggers inflammatory responses via Toll-like receptor pathways, contributing to hepatic inflammation and fibrosis characteristic of MASLD [47,48]. Studies have identified a link between metabolic dysfunction and lower levels of bacteria that produce short-chain fatty acids (SCFAs), particularly propionate and butyrate [49]. Dysbiosis can also influence bile acid metabolism and SCFA production, both of which are critical regulators of lipid and glucose metabolism. Disruptions in these metabolic pathways may exacerbate insulin resistance (IR) and promote hepatic steatosis, further advancing MASLD progression [50].

IR plays a central role in the pathogenesis of MASLD, with evidence suggesting it is essential for disease development and progression [51–53]. IR is characterised by impaired insulin signalling in the adipose tissue, leading to suppression of lipolysis and increased release of free fatty acids circulating to the liver, promoting hepatic lipid accumulation. The excess lipid species such as diacylglycerols and ceramides activate stress-related signalling pathways, including protein kinases and c-Jun N-terminal kinase, which further impair insulin signalling and exacerbate metabolic dysfunction [54]. This excessive hepatic lipid deposition triggers oxidative stress, endoplasmic reticulum stress, and inflammation, ultimately contributing to fibrosis and liver dysfunction. Clinical studies reinforce the strong association between IR and MASLD, with research indicating that two-thirds of individuals with type 2 diabetes have MASLD and that IR is a significant predictor of the disease in both lean and obese populations [55]. Although some studies report MASLD in the absence of IR, these cases often show milder histological features [56]. Overall, while IR is not the sole determinant of MASLD, its role in hepatic lipid metabolism and inflammation makes it a key therapeutic target for disease management.

#### Diagnosis and biomarkers

The diagnosis of MASLD has evolved with advancements in non-invasive techniques and biomarker research. Traditional imaging modalities like ultrasound and MRI remain foundational for detecting hepatic steatosis. Interestingly, multiparametric MRI has demonstrated cost-effectiveness in diagnosing and managing MASLD, reducing the need for liver biopsies and enhancing diagnostic certainty [57]. There is increasing use of serum biomarkers and non-invasive diagnostic techniques for MASLD. Biomarkers such as Fatty Liver Index [58], Hepatic Steatosis Index [59], Liver Fat Score [60], Steatotest [59], and controlled attenuation probes [61] have been used to detect liver fat. However, liver biopsy remains the gold standard diagnostic tool, as it can provide important prognostic information concerning the extent of fibrosis and severity of inflammation [62,63].

#### Therapeutic strategies

MASLD is a complex condition requiring a multifaceted therapeutic approach. Current management strategies primarily focus on lifestyle interventions, exercise, and weight loss, which have been shown to improve liver histology and reduce disease progression [64,65].

In pharmacological therapy, recent FDA approvals have marked major progress. Semaglutide, a GLP-1 receptor agonist originally developed for T2D and obesity, was recently approved for the treatment of noncirrhotic MASH with moderate to advanced fibrosis. In a phase 3 trial, semaglutide achieved MASH resolution in 63% of participants compared with 34% in the placebo group, and fibrosis regression in 37% compared with 22% in the placebo group, after 72 weeks of treatment [66]. Earlier studies also confirmed that GLP-1 agonists reduce liver fat content and improve liver enzymes in MASLD and MASH [67,68]. Similarly, resmetirom (Rezdiffra), a thyroid hormone receptor-β agonist, became the first drug approved for MASH with moderate to advanced fibrosis, as a phase 3 trial showed resolution of MASH without fibrosis worsening in 25.9%–29.9% of patients compared with 9.7% on placebo [69].

In addition to recently approved therapies, fibroblast growth factor 21 (FGF21) agonists represent another avenue of therapeutic exploration. FGF21 plays a critical role in regulating lipid metabolism and insulin sensitivity. Early-phase clinical trials have indicated that FGF21 analogues (pegbelfermin, efruxifermin, or pegozafermin) can reduce liver fat content and improve markers of liver injury in individuals with MASLD [70–72]. Additionally, peroxisome proliferator-activated receptor (PPAR) agonists, such as pioglitazone, pemafbrate and lanifibranor, are being studied for their potential to modulate lipid metabolism, inflammation, and fibrosis in MASLD patients [73–76]. These agents have shown promise in improving liver histology, with further clinical trials ongoing to confirm their long-term efficacy and safety profiles.

Together, these advances signal a new era in MASLD treatment, where lifestyle modification remains the foundation of care but is increasingly complemented by targeted pharmacological therapies. Importantly, while semaglutide and resmetirom have now demonstrated clinical efficacy and received regulatory approval for selected patients with MASH, several other agents, including FGF21 and PPAR agonists, remain under clinical investigation and require further validation in phase 3 trials.

### Iron and MASLD

Approximately one-third of MASLD patients exhibit biochemical evidence of altered iron metabolism, commonly reflected in elevated serum ferritin levels [6]. A subset of these patients will have DIOS. DIOS is characterised by mild to moderate hepatic iron accumulation in the absence of genetic mutations typically associated with hereditary haemochromatosis [77]. The presence of excess iron in MASLD patients is believed to exacerbate liver injury by generating reactive oxygen species (ROS), leading to oxidative stress, lipid peroxidation, and hepatocellular damage [78–80].

Several studies have found a positive correlation between iron parameters and more advanced stages or higher incidence of MASLD [81,82]. Serum ferritin, a major iron storage protein, has been shown to correlate positively with disease progression [83]. A large study involving 25 597 Korean MASLD patients identified serum ferritin as an independent predictor of liver steatosis and fibrosis [84]. Similarly, a US-based study concluded that serum ferritin could help identify MASLD patients at risk of developing MASH and advanced fibrosis [85]. However, conflicting findings exist. A small study involving 30 MASLD/MASH patients reported no significant correlation between serum ferritin and fibrosis but this finding is likely due to limited sample size and lower mean BMI [86].

#### Mechanistic insights

There is increasing evidence that elevated hepatic iron stores can contribute to fibrosis and necroinflammation in MASLD. Iron generates ROS via the Fenton reaction, leading to oxidative damage and the production of pro-inflammatory cytokines [78]. Hepatic iron appears to play a role in MASH pathogenesis by activating an oxidative stress cascade in the liver [78]. Oxidative stress promotes cell injury and death, and it can cause damage to DNA, proteins and lipids within the hepatocytes of NASH patients [79]. 7,8-dihydro-8-oxo-2′-deoxyguanosine (8-oxodG), a major product of DNA oxidation, was found to be significantly related to iron overload, and its levels were reduced after venesection in MASH patients [80].

The detrimental effects of iron overload in MASLD underscore the importance of regulatory mechanisms that maintain iron homeostasis. Hepcidin, the central regulator of systemic iron balance, plays a crucial role in this process by inhibiting FPN, the only known iron exporter, thereby controlling dietary iron absorption and its distribution in tissues. Hepcidin expression is often dysregulated in MASLD.

In the past two decades, several researchers have sought to determine the expression of hepcidin in MASLD, and has been the subject of considerable debate. To determine the level of serum iron markers in patients with several chronic diseases, Radicheva et al. analysed the serum samples of 22 MAFLD patients with 60 healthy controls [83]. The findings reported an increase in serum iron, ferritin, and transferrin saturation and a decrease in serum hepcidin in patients with MASLD compared with the control group. Interestingly, they also found a further decrease in serum hepcidin in patients with MASH (*n* = 16) compared with those with MASLD. The major limitation of the present study is the small sample size. Similarly, researchers have previously shown that Hfe^−/−^ mice fed a high-calorie diet developed MASH and had reduced hepatic hepcidin mRNA expression compared with controls [87]. However, other researchers disagree with this notion, as they argue that hepcidin expression is increased in MASLD patients [88,89]. Of interest, a study that investigated the putative pathways underlying iron accumulation in MASLD patients reported that the hepatic expression of hepcidin increased in MASLD patients with progressive iron accumulation [89]. A key limitation of that work, however, was the absence of BMI data, making it difficult to separate the influence of obesity. It is likely that inflammatory cytokines and adiposity-related factors contribute to hepcidin up-regulation in this setting, complicating interpretation.

There is increasing evidence that serum hepcidin levels may reflect adipose tissue mass more than liver histology severity, as adipocytes themselves are capable of synthesising hepcidin [90,91]. A study examining the relationship between BMI, liver histology, and serum hepcidin discovered a strong positive correlation between obesity and serum hepcidin levels, independent of liver disease [90]. Similarly, hepcidin expression is higher in severe obesity compared with mild obesity [92]. One possible explanation for this phenomenon is the elevated levels of leptin, an appetite-suppressing adipokine, in obese patients. Researchers have found that leptin can induce hepatic hepcidin expression via the Jak2/STAT3 signalling pathway [93]. Therefore, it is plausible that obesity and an increase in AT mass, rather than the presence of MASLD, are associated with hepcidin up-regulation.

#### Therapeutics

Hepcidin has shown therapeutic potential in MASLD by modulating hepatic iron levels and metabolic processes. Minihepcidin (PR65) administration in iron-loaded, hepcidin-deficient mice redistributed iron from the liver to the spleen, suggesting a role in mitigating MASLD progression [94,95]. Additionally, Tmprss6 knockout mice, which exhibit increased hepcidin levels, were protected from diet-induced obesity, hepatic steatosis, and IR, highlighting hepcidin’s metabolic influence [94]. Further, overexpression of hepcidin using rAAV2/8-Hamp alleviated steatohepatitis and fibrosis in a diet-induced MASH model by reducing hepatic fat accumulation, inflammation, and fibrogenesis [96]. These findings suggest hepcidin as a potential therapeutic agent in MASLD.

Venesection has also been investigated as a therapeutic option for MASLD patients with elevated iron stores, given its potential to improve liver enzymes, IR (HOMA-IR), and lipid profiles. A single-arm study reported that iron depletion through venesection improved hepatic steatosis and hepatocyte ballooning, suggesting a beneficial effect of lowering systemic iron burden [97]. However, evidence remains inconclusive [98]. Randomised controlled trials have produced conflicting results—while one study reported improvements in hepatic steatosis with venesection, there was no significant effect on lobular inflammation or hepatocyte ballooning [99]. Another randomised controlled trial by Adams et al. found that reducing ferritin through phlebotomy did not improve hepatic fat content, IR, or liver enzymes in MASLD patients [100]. Therefore, although iron reduction remains a mechanistically appealing approach, routine use of venesection in MASLD cannot yet be justified without stronger, consistent evidence from large, well-designed clinical trials.

The impact of iron overload in MASLD underscores how metabolic and environmental factors can converge to accelerate liver injury. Alcohol consumption represents another key driver of oxidative stress, inflammation, and fibrogenesis, with mechanistic pathways that often overlap with those seen in metabolic dysfunction and iron dysregulation. These shared injury pathways are integrated in Figure 2. Understanding the pathophysiology of ALD is therefore critical to appreciating the additive or synergistic effects that can occur when these insults co-exist.

### Alcohol-associated liver disease

ALD is among the leading causes of chronic liver disease worldwide, accounting for an estimated 40%–50% of liver-related deaths [101,102]. Like MASLD, ALD also encompasses a spectrum from simple alcoholic fatty liver (steatosis) to alcohol-associated steatohepatitis, fibrosis/cirrhosis, and even HCC [103]. ALD progresses through overlapping stages. Early fatty liver is often asymptomatic and reversible with abstinence. Persistent heavy drinking may lead to alcoholic steatohepatitis, characterised by steatosis with inflammation, which can manifest chronically or acutely as alcoholic hepatitis (AH), presenting with jaundice, fever and abdominal pain [104]. The risk of advanced ALD is strongly dose-dependent, yet only a minority (10%–30%) of chronic heavy drinkers ever develop cirrhosis [105]. This indicates that host factors (genetics, sex) and co-morbid conditions (obesity, viral hepatitis) modulate individual susceptibility.

#### Pathogenesis

Chronic alcohol ingestion leads to liver injury through multiple interrelated mechanisms. Hepatocytes metabolise ethanol via alcohol dehydrogenase and CYP2E1, generating acetaldehyde and ROS. Excess ROS production in chronic drinking overwhelms antioxidant defences, causing oxidative stress [106]. This triggers lipid peroxidation and formation of reactive aldehyde-protein adducts, leading to hepatocellular damage and neoantigens that provoke immune responses. In addition to ROS production, ethanol metabolism increases the hepatic NADH/NAD^+^ ratio, disrupting mitochondrial redox homeostasis and impairing β-oxidation of fatty acids, thereby promoting hepatic steatosis [107]. Additionally, alcohol-induced hepatocyte injury and gut-derived endotoxins activate Kupffer cells and recruit neutrophils, resulting in an inflammatory cascade. Alcoholic steatohepatitis is characterised by infiltration of neutrophils and macrophages in the liver lobule [103,108], accompanied by elevated proinflammatory cytokines. Adaptive immunity may also be altered, especially in severe AH, further amplifying hepatocyte death and fibrogenesis [105]. Furthermore, excessive alcohol disrupts the intestinal barrier and alters the gut microbiome. This allows endotoxin to translocate to the liver via the portal circulation, where it activates Toll-like receptors on Kupffer cells and other innate immune cells [109]. Gut dysbiosis also contributes to systemic inflammation and increases susceptibility to liver injury.

#### Diagnosis and treatment

Diagnosis of ALD requires a history of significant alcohol use and exclusion of other liver diseases. No single test confirms ALD, but characteristic features include moderate elevations in aminotransferases (typically <300 U/L), with AST often more elevated than ALT (AST:ALT >2) [110]. GGT is frequently elevated in chronic drinkers, while serum bilirubin and INR rise in severe disease due to impaired liver function. Imaging (ultrasound, transient elastography) aids in assessing steatosis and fibrosis. Non-invasive fibrosis scores (FIB-4, APRI) are also used [111,112]. Liver biopsy, though invasive, remains the gold standard and shows macrovesicular steatosis, ballooning, Mallory–Denk bodies, neutrophilic infiltration, and pericentral fibrosis.

The cornerstone of ALD management is alcohol abstinence, which can halt progression and improve survival [113,114]. Sustained abstinence often requires a multidisciplinary approach, including counselling, support groups, and pharmacotherapy. Nutritional support is essential, as malnutrition is common; a high-protein diet with supplements such as thiamine, folate, and zinc is recommended [115]. Medical therapy is reserved for severe AH. Prednisolone (40 mg/day for 28 days) is the first-line treatment [116,117], improving short-term survival, but must be used cautiously due to infection risk. For end-stage ALD, liver transplantation offers ∼70% 5-year survival in well-selected candidates. While a 6-month abstinence period was once standard, early transplantation in patients with acute AH is now considered in some centres in carefully selected cases [118].

#### Emerging therapies

Given that no FDA-approved drug specifically targets ALD pathogenesis, there is intense research into new treatments. Recent and ongoing clinical trials are exploring several promising avenues. One major area of investigation involves anti-inflammatory and immune-modulating agents [119]. These therapies aim to reduce liver injury by targeting key inflammatory pathways without compromising host defence. Interleukin-1 (IL-1) inhibitors are being studied to block IL-1β signalling, as inhibiting IL-1 in mice showed improved hepatocyte inflammation and caused hepatic regeneration [120,121]. Pan-caspase inhibitors are designed to prevent hepatocyte apoptosis and have been shown to reduce fibrosis in mice [122], while inhibitors of apoptosis signal-regulating kinase 1 target stress-related pathways implicated in disease progression [119]. Additionally, CCR2/CCR5 antagonists are under investigation for their ability to block monocyte recruitment to the liver, thereby mitigating immune-mediated damage [123].

Another promising frontier lies in gut microbiome-directed therapies, based on the pivotal role of the gut–liver axis in ALD pathogenesis. Strategies such as probiotics, prebiotics, and fecal microbiota transplantation (FMT) are being used to restore intestinal barrier integrity and microbiota composition. Preliminary studies have shown that FMT can improve survival in patients with AH compared with standard care [124,125], although it can also cause infection. Other approaches under evaluation include oral antibiotics and engineered probiotics designed to reduce endotoxin-producing bacterial populations [106].

Regenerative and antifibrotic therapies are also gaining momentum. Granulocyte colony-stimulating factor has shown encouraging results in early trials by promoting bone marrow stem cell mobilisation and enhancing hepatocyte regeneration, leading to improved survival in acute AH [126]. Similarly, growth factors such as interleukin-22 (IL-22) are under clinical evaluation for their regenerative and cytoprotective effects on hepatocytes [127].

Collectively, these approaches highlight the expanding therapeutic landscape in ALD. However, most remain investigational, with evidence largely derived from preclinical studies, early-phase clinical trials, or small patient cohorts. At present, alcohol abstinence, nutritional support, and management of alcohol-related complications remain the cornerstone of clinical care. Larger randomised clinical trials are needed to establish the efficacy and safety of anti-inflammatory, microbiome-directed, regenerative, and antifibrotic therapies before they can be widely adopted in clinical practice.

While the pathogenic effects of alcohol on the liver are well established, accumulating evidence suggests that iron overload is a frequent and clinically significant cofactor in ALD. By compounding oxidative stress, mitochondrial injury, and inflammatory responses, excess iron can amplify alcohol-induced hepatotoxicity and accelerate disease progression. Understanding this interplay between alcohol and iron is therefore critical for identifying high-risk patients and developing targeted therapeutic strategies.

### Iron and ALD disease

ALD often features excess iron in the liver, which can worsen alcohol-induced injury. In fact, up to half of ALD patients display hepatic iron overload, a factor linked to more rapid disease progression, higher risk of HCC, and poorer survival [7,8]. Iron accumulation in ALD results from dysregulated iron homeostasis, as ethanol exposure suppresses the iron-regulatory hormone hepcidin (leading to increased intestinal iron absorption) and up-regulates transferrin receptor-1 on hepatocytes (enhancing iron uptake) [128,129]. Unlike hereditary haemochromatosis, classical HFE gene mutations are typically absent from ALD; instead, alcohol-induced oxidative stress and elevated erythropoietin contribute to low hepcidin levels and iron overload [130]. The net effect is excessive iron deposition in both parenchymal hepatocytes and Kupffer cells, compounding liver injury.

#### Pathogenesis

Excess iron catalyses the formation of ROS via Fenton chemistry, converging with ethanol metabolism–derived ROS to amplify oxidative stress [131]. This heightened oxidative stress causes peroxidation of lipids, proteins, and DNA in hepatocytes, driving cell injury [130]. One major target is the mitochondrion; studies in rat models show that chronic alcohol plus iron intake leads to significant mitochondrial DNA damage and impaired respiratory chain function, whereas alcohol alone is less injurious, as shown in Figure 2 [132]. Iron-induced mitochondrial dysfunction further increases ROS generation and decreases ATP synthesis, exacerbating hepatocellular damage.

Iron overload in ALD not only causes non-specific oxidative damage but also triggers ferroptosis—a regulated necrotic cell death driven by iron-dependent lipid peroxidation. Ferroptosis has emerged as an important mechanism in ALD pathogenesis [133]. In experimental models, chronic ethanol exposure induced clear ferroptotic changes in the liver, evidenced by increased labile iron, lipid peroxidation, and depletion of glutathione peroxidase activity [134]. Hepatocytes undergoing ferroptosis display lethal accumulation of lipid ROS, which can be mitigated by iron chelators or antioxidants. Notably, pharmacologic inhibitors of ferroptosis (ferrostatin-1) significantly alleviated alcohol-induced liver injury in mice [135], underscoring that ferroptosis contributes to ALD and highlighting it as a potential therapeutic target.

Iron aggravates the inflammatory cascade in ALD. Iron-laden hepatocytes produce excess ROS and may release damage-associated molecular patterns (DAMPs) upon injury, which activate Kupffer cells [136]. Iron overload directly stimulates Kupffer cells to secrete pro-inflammatory cytokines and chemokines, fuelling hepatic inflammation [7]. In particular, DAMPs from ferroptotic hepatocytes can trigger the NLRP3 inflammasome in Kupffer cells, amplifying interleukin and TNFα release [137,138]. Concurrently, iron and ROS activate hepatic stellate cells either directly or via Kupffer cell signals, driving these fibrogenic cells to produce collagen and extracellular matrix [139]. Persistently activated stellate cells lead to accelerated fibrosis and cirrhosis progression in ALD [131,140]. Thus, excess iron creates a pro-inflammatory, pro-fibrotic microenvironment in the alcohol-injured liver, linking iron overload to more severe hepatitis and scarring (see Figure 2) [130].

#### Clinical implications and therapeutic perspectives

Clinically, the presence of iron overload in ALD often signifies more advanced disease. Serum markers of iron overload (elevated ferritin, high transferrin saturation) are frequently observed in ALD patients [141], and inappropriately low hepcidin levels correlate with greater iron loading. Importantly, low hepcidin has been associated with worse long-term outcomes in alcoholic cirrhosis [142], suggesting that dysregulated iron metabolism contributes to disease severity. These findings raise interest in iron-related biomarkers for risk stratification—for example, ferritin and hepcidin levels might help gauge prognosis or response to therapy. From a therapeutic standpoint, targeting iron is a logical strategy. Phlebotomy or iron chelators could reduce hepatic iron stores, and although routine phlebotomy in ALD is not yet standard, trials are exploring its impact on outcomes like fibrosis and cancer risk [143]. It will also be important to determine whether newer iron-directed therapeutics, including hepcidin mimetics or FPN inhibitors, may have a therapeutic role in ALD. Moreover, the success of ferroptosis inhibitors in preclinical models hints at new treatments that interrupt iron's pathogenic cascade. In summary, iron plays a pivotal role in ALD's pathogenesis—promoting oxidative stress, mitochondrial damage, ferroptosis, and inflammation—and it stands as both a marker of disease severity and a promising target for adjunctive therapy.

The pathogenic synergy between alcohol and iron illustrates how multiple liver injury pathways can converge to amplify disease severity. This concept extends beyond iron overload to other co-existing insults, most notably the combination of metabolic dysfunction and alcohol intake now recognised as MetALD, a phenotype that is increasingly gaining attention in clinical and research settings.

### MASLD and increased alcohol intake (MetALD)

MetALD is a newly defined entity describing individuals with MASLD who also consume moderate amounts of alcohol. Patients with MetALD typically exhibit features of metabolic syndrome and a history of at least moderate alcohol consumption (see Figure 1). The combination of metabolic dysfunction and alcohol intake additively accelerates the progression to steatohepatitis, fibrosis, and HCC [144]. For instance, obese drinkers have 2–3 times higher risk of advanced fibrosis or cirrhosis compared with lean drinkers [145,146], and moderate alcohol intake in type 2 diabetics with NAFLD markedly raises the odds of progression to advanced liver disease [147]. Additionally, because both metabolic dysfunction and alcohol injure the liver, MetALD is often an aggressive phenotype. Studies indicate that MetALD patients tend to have higher levels of ALT and AST than metabolically driven MASLD alone [148,149], and especially elevated γ-glutamyltransferase (GGT), reflecting the added oxidative stress from alcohol [3].

#### Pathogenesis

The mechanisms underlying MetALD involve an interplay of the pathways from MASLD and ALD, which together produce an additive—often synergistic—“two-hit” (or more) injury to the liver [150]. Both metabolic syndrome and chronic alcohol consumption cause hepatic fat accumulation and lipotoxicity, triggering oxidative stress, endoplasmic reticulum stress, mitochondrial dysfunction, and hepatocellular injury with inflammation and fibrogenesis [2,144]. Interestingly, studies have shown that metabolic dysfunction lowers the liver’s tolerance to alcohol as obesity and IR impair alcohol metabolism and clearance [151]. Notably, patients with metabolic fatty liver often have reduced activity of alcohol dehydrogenase (ADH) and aldehyde dehydrogenase (ALDH) [151,152], the enzymes that detoxify ethanol and its metabolite acetaldehyde. This means even moderate drinking can lead to disproportionately high acetaldehyde exposure in the liver, exacerbating cell injury. Experimental studies support this, showing that obese or diabetic rodents exhibit significantly reduced ADH activity and slower ethanol clearance compared with lean controls [153,154].

Furthermore, alcohol may further disrupt metabolic pathways by interfering with retinoic acid (RA) synthesis, a molecule essential for hepatic lipid homeostasis and shown to exert protective effects in MASLD [155]. Specifically, alcohol inhibits ADH-mediated oxidation of retinol to retinal, a critical step in RA biosynthesis, thereby impairing RA availability and its lipid-regulating functions [156]. Alcohol also induces CYP2E1, which generates ROS, and in insulin-resistant states, CYP2E1 induction is sustained, fuelling a cycle of oxidative damage and inflammation [157]. Other shared pathways include gut-derived endotoxin; both heavy drinking and obesity lead to gut microbiome changes and increased intestinal permeability.

Additionally, genetic modifiers (such as the PNPLA3 I148M variant) predispose individuals to more severe steatosis and fibrosis in both alcohol- and metabolic-related fatty liver; patients carrying such risk alleles are likely especially vulnerable in the setting of MetALD. Metabolic syndrome sensitises the liver to alcohol’s toxicity (by hampering detoxification and antioxidant defences), while alcohol intake accelerates the metabolic injury (by adding oxidative stress, empty calories, and impairing lipid metabolism). This bidirectional synergy explains why MetALD often progresses more rapidly to steatohepatitis, advanced fibrosis, or HCC than MASLD or ALD alone.

#### Diagnosis, prognosis, and management of MetALD

The diagnostic work-up for MetALD mirrors that of other fatty liver diseases and includes measurement of liver enzymes and biochemical markers of liver function (bilirubin, INR, and albumin concentrations), exclusion of alternative or co-existing liver diseases, and assessment of long-term risk of adverse outcomes. Risk stratification often begins with non-invasive fibrosis scores such as FIB-4, with or without additional testing by FibroScan or the Enhanced Liver Fibrosis test. Imaging modalities such as ultrasound also support evaluation, while liver biopsy is indicated when diagnosis remains uncertain or fibrosis requires definitive staging. Histology typically reveals steatosis with overlapping features of both MASLD and ALD. A major challenge lies in accurately quantifying alcohol intake, as thresholds vary internationally and self-reporting is often unreliable [158]. Tools such as structured questionnaires, collateral history, and biomarkers (e.g. phosphatidylethanol, carbohydrate-deficient transferrin) can help uncover clinically significant alcohol use [159–161]. Clinicians should maintain a high index of suspicion for MetALD in any steatotic patient who is not a confirmed abstainer.

Recognising MetALD carries critical prognostic and therapeutic implications. Patients with both metabolic dysfunction and alcohol exposure have worse outcomes than those with MASLD or ALD alone, including a higher risk of cirrhosis, hepatic decompensation, CVD, malignancy, and HCC [145,146,148,162,163]. A large cohort study (*n* ≈ 12 600) demonstrated that while both metabolic dysfunction and excessive alcohol use independently increase mortality risk, having both factors conferred the highest risk of all [163]. Even moderate alcohol intake in the context of metabolic risk has been shown to accelerate liver disease progression and increase HCC incidence [162].

Management must be multifaceted. Core strategies include lifestyle modification (weight loss, exercise, Mediterranean or low-carb diets), optimal control of diabetes and lipids, and complete alcohol cessation. Current guidelines recommend abstinence even in cases of “moderate” drinking, as binge episodes (even if infrequent) can significantly worsen prognosis [164,165]. Pharmacological agents under investigation for MASH (e.g. FXR agonists, GLP-1 analogues, and THR-β agonists) may offer promise, though MetALD patients have historically been excluded from such trials. Likewise, standard treatments for alcohol use disorder (e.g. motivational therapy, and naltrexone, acamprosate) should be integrated into care.

Although MetALD represents a high-risk condition, it is also highly modifiable. Sustained weight loss and alcohol abstinence have both been shown to improve liver histology and reduce progression to cirrhosis [166]. Going forward, early identification and intervention, combined with broader public health efforts targeting alcohol misuse and obesity, will be essential to reduce the growing burden of MetALD.

While the dual insult of metabolic dysfunction and alcohol intake already drives an aggressive disease course, the addition of iron overload may represent an even more injurious “third hit.” Evidence from MASLD and ALD models suggests that excess hepatic iron can potentiate oxidative stress, and accelerate fibrosis, mechanisms that are likely to operate in MetALD as well. This emerging triple-hit paradigm, though understudied, warrants closer examination to understand its role in shaping disease severity and progression.

### Iron and MetALD (Fe-MetALD)

Iron plays a dual role in liver physiology, supporting vital metabolic processes at physiological levels, but becoming highly toxic when dysregulated. While its individual contributions to MASLD and ALD are well studied, iron's role in the overlapping condition of MetALD remains largely unexplored. This “triple-hit” scenario, where iron overload, metabolic dysfunction, and sustained alcohol exposure converge, may represent a particularly aggressive liver disease phenotype, yet remains a significant gap in hepatology research. This proposed overlap phenotype is summarised conceptually in Figure 1. Key distinguishing features of Fe-MetALD compared with MASLD, ALD and MetALD are summarised in Table 1.

#### Mechanistic insights

The hepatotoxic synergy of these three insults is biologically plausible. Both metabolic syndrome and alcohol independently promote hepatic lipid accumulation and oxidative stress through distinct but converging pathways [34,40,106]. Excess iron further amplifies this injury via the Fenton reaction, generating highly reactive hydroxyl radicals that intensify oxidative damage to lipids, proteins, and DNA [78]. This convergence sets the stage for a potent “triple-hit” model of liver injury characterised by heightened inflammation, mitochondrial dysfunction, impaired autophagy, and fibrogenesis. The integrated molecular framework for this proposed Fe-MetALD phenotype is illustrated in Figure 2.

In a study modelling this “triple-hit” scenario, Hfe^−/−^ mice fed a high-fat diet and given ethanol for eight weeks developed severe steatohepatitis, significant fibrosis, and increased apoptosis, with pathology exceeding that observed in models exposed to only one or two insults [87]. Mechanistically, the triple-hit model exhibited heightened endoplasmic reticulum stress, suppressed autophagy, and defective protein quality control, indicating that iron impairs the hepatocyte’s ability to withstand metabolic and alcohol-induced stress [87].

Similarly, Tsukamoto et al. provided early experimental evidence for the triple-hit hypothesis using a high-fat diet (HFD) model [167]. Rats fed HFD in combination with both ethanol and iron developed far worse outcomes than those receiving HFD with ethanol or HFD with iron alone. After 16 weeks, the triple-hit group showed markedly higher levels of lipid peroxidation products (MDA and 4-HNE), together with elevated ALT and AST, indicating more severe hepatocellular injury. Histologically, this combined insult exacerbated hepatocyte damage, promoted fibrogenesis, and even produced cirrhosis in some animals, changes not as severe in the dual-hit groups [167]. These findings underscore that when fat, alcohol, and iron converge, the resulting liver injury is more aggressive than any dual combination.

Additionally, MetALD appears to synergistically worsen liver injury by disrupting iron metabolism, increasing inflammation, and activating hypoxia signalling pathways [168]. Rats on combined diets with HFD and alcohol developed greater hepatocyte ballooning and inflammation than those on HFD alone, accompanied by increased hepatic iron deposition and depletion of circulating iron. Liver expression of ferritin and FPN was up-regulated, indicating altered iron handling, and there was nuclear ferritin accumulation in hepatocytes with activation of HIF-1α and STAT3—a marker of oxidative stress [168]. These findings suggest that metabolic dysfunction and alcohol impair systemic iron handling, allowing toxic hepatic iron build-up that potentiates cellular damage.

While the literature directly addressing Fe-MetALD is sparse, the additive pathology is supported by extensive evidence from MASLD and ALD studies. In MASLD, iron overload, often presenting as DIOS, is associated with increased liver injury, fibrosis progression, and higher risk of HCC [6]. Similarly, in ALD, hepatic iron accumulation correlates with greater necroinflammation and fibrosis [130]. Given this, it is plausible that MetALD patients with concurrent iron overload experience more severe disease trajectories, yet this hypothesis remains insufficiently tested in clinical settings.

The regulatory hormone hepcidin adds further complexity. In MASLD, hepcidin levels are variably altered, up-regulated in obesity and inflammation, but paradoxically down-regulated in advanced disease or iron overload [83,91]. Alcohol independently suppresses hepcidin expression via oxidative stress and inhibition of the BMP/SMAD pathway [169,170], possibly compounding iron dysregulation in MetALD. This dysregulation likely facilitates iron retention in hepatocytes and Kupffer cells, fuelling further oxidative injury.

#### Clinical implications & research gaps

Clinically, this raises concern for MetALD patients with concurrent iron-loading conditions such as HFE mutations or elevated ferritin. Despite no formal diagnostic criteria for Fe-MetALD, assessing iron indices in these patients could improve risk stratification and inform management. Serum ferritin has already been shown to function as an independent predictor of mortality and adverse clinical events in cirrhotic patients awaiting liver transplantation, with higher ferritin levels identifying individuals at substantially increased risk [171].

Consequently, iron-lowering strategies, such as phlebotomy or hepcidin mimetics, have emerged as potential therapeutic approaches for Fe-MetALD alongside established interventions, including alcohol cessation and weight loss. Although phlebotomy, iron chelation, hepcidin mimetics, and FPN-modulating approaches are biologically plausible strategies, clinical evidence supporting their routine use in MASLD, ALD, or MetALD remains limited. Nevertheless, preclinical studies have demonstrated that TMPRSS6 inhibition and hepcidin gene therapy can reduce hepatic steatosis, inflammation, and fibrosis, supporting further investigation of iron-targeted interventions as potential therapies for Fe-MetALD [94,96].

However, major research gaps remain. First, the long-term impact of mild iron overload in the context of MetALD, particularly in patients with moderate alcohol consumption, obesity, and HFE mutations, has not been adequately studied. Second, there is a lack of human data explicitly linking iron overload with clinical outcomes in MetALD. Prospective studies are needed to explore whether patients with “triple-hit” profiles have accelerated progression to cirrhosis or HCC. Additionally, existing non-invasive scoring systems do not incorporate iron biomarkers, despite their prognostic potential. Integration of serum ferritin or transferrin saturation into tools like FIB-4 or NAFLD fibrosis score could improve sensitivity in MetALD cases.

Fe-MetALD represents a biologically plausible and clinically relevant disease framework that warrants greater scientific attention. Current data, although limited, suggest that the interplay of iron, metabolic dysfunction, and alcohol produces a uniquely harmful liver environment, promoting oxidative stress, inflammation, mitochondrial dysfunction, and impaired tissue repair. Identifying and managing this “triple-hit” phenotype could have significant implications for patient outcomes. Targeted mechanistic studies, biomarker development, and clinical trials should explicitly include iron as a cofactor in MetALD to unlock new therapeutic avenues. At present, most iron-directed interventions remain experimental in SLD, with supporting evidence derived primarily from preclinical models and a limited number of early-phase clinical studies. Therefore, future research is required to establish whether iron-directed therapies can provide meaningful clinical benefit beyond established interventions targeting metabolic dysfunction and alcohol exposure.

The key clinical, pathological and mechanistic distinctions between MASLD, ALD, MetALD, and the proposed Fe-MetALD phenotype are summarised in Table 1.

## Conclusion

SLD now encompasses three major phenotypes: MASLD, ALD, and their overlap, MetALD. Both metabolic dysfunction and alcohol consumption induce similar histopathological changes, including steatosis, inflammation, hepatocyte injury, and fibrosis, and when present together, these factors often act additively or synergistically to worsen liver injury. Emerging evidence suggests that a “third hit” from iron overload in MetALD (Fe-MetALD) may drive an even more aggressive disease course, amplifying oxidative stress, mitochondrial injury, and fibrogenesis. Although few studies have directly compared the impact of this triple-hit to the dual-hit scenarios, preliminary data indicate markedly worse outcomes when all three insults converge. Patients with Fe-MetALD likely represent a high-risk subgroup in whom disease progression can be rapid and clinically silent unless all contributing factors—metabolic dysfunction, alcohol use, and iron overload—are identified and addressed early.

As illustrated in Figures 1 and 2, Fe-MetALD may represent a biologically distinct triple-hit phenotype in which metabolic dysfunction, alcohol exposure and iron overload interact to exacerbate liver injury and disease progression. Closing the current knowledge gaps through dedicated mechanistic and translational studies will be critical to defining the true burden of Fe-MetALD and informing targeted prevention and treatment strategies.

## Open Access

Open access for this article was enabled by the participation of the University of Queensland in an all-inclusive Read & Publish agreement with Portland Press and the Biochemical Society under an agreement with CAUL.

## Abbreviations

AH: alcoholic hepatitis
ALD: alcohol-associated liver disease
CVD: cardiovascular disease
DAMPs: damage-associated molecular patterns
DIOS: dysmetabolic iron overload syndrome
DMT1: divalent metal transporter 1
FGF21: fibroblast growth factor 21
FMT: fecal microbiota transplantation
FPN: ferroportin
GGT: γ-glutamyltransferase
HFD: high-fat diet
HJV: hemojuvelin
IR: insulin resistance
IL-1: interleukin-1
MASH: metabolic dysfunction-associated steatohepatitis
MASLD: metabolic dysfunction-associated steatotic liver disease
MetALD: metabolic dysfunction and alcohol-associated liver disease
NASH: non-alcoholic steatohepatitis
PPAR: peroxisome proliferator-activated receptor
ROS: reactive oxygen species
RA: retinoic acid
SCFAs: short-chain fatty acids
SLD: steatotic liver disease
TFR: transferrin receptors
TFR2: transferrin receptor 2
